# Explorations of avoidance and approach coping and perceived stress with a computer-based avatar task: detrimental effects of resignation and withdrawal

**DOI:** 10.7717/peerj.11265

**Published:** 2021-04-15

**Authors:** M. Todd Allen

**Affiliations:** School of Psychological Sciences, University of Northern Colorado, Greeley, CO, United States of America

**Keywords:** Approach, Avoidance, Coping styles, Computer-based task, Perceived stress

## Abstract

**Background:**

Individuals differ in how they react to stress or trauma through different coping styles in which they may deal directly with a stressor by adopting approach coping styles or disengage with a stressor by utilizing avoidant coping styles. Avoidant coping styles have been linked to adverse outcomes including psychological distress, anxiety disorders, and post-traumatic stress disorder (PTSD). Recently, avoidance coping styles as measured by a subset of items on the Brief COPE were found to have a weak positive relationship with performance on a computer-based avatar task which is related to avoidant personality temperaments. This avatar task was developed as an alternative for paper and pencil self-report inventories for measuring avoidant tendencies based on possible response biases of avoidant individuals. In the current study, avoidance and approach coping styles as measured by the Brief Approach/Avoidance Coping Questionnaire (BACQ) were compared to avoidant coping as measured by the Brief COPE and performance on the avatar task. In addition to approach and avoidance coping, the BACQ also measures active avoidance coping (i.e., diversion) and passive avoidance coping (i.e., resignation and withdrawal). The relationships between approach and avoidance coping and performance on the avatar task were also analyzed with the outcome of perceived stress as measured by the Perceived Stress Scale (PSS).

**Methods:**

One hundred undergraduates voluntarily completed the BACQ, the Brief COPE, and the PSS. Participants also completed a computer-based task in which they guided an avatar through a series of social situations where they indicated how they would interact with or avoid interacting with strangers.

**Results:**

Approach coping had a weak negative relationship to avoidance coping as measured by the BACQ and the Brief COPE. Performance on the avatar task had a moderate positive relationship with avoidance coping (diversion as well as resignation and withdrawal) as measured by the BACQ and a moderate negative relationship with approach coping as measured by the BACQ. A model including only approach, diversion, and resignation and withdrawal coping best predicted performance on the avatar task in a linear regression model. While resignation and withdrawal coping and diversion coping had moderate positive relationships to avatar task scores, only resignation and withdrawal had a strong positive relationship to perceived stress. A model than included only resignation and withdrawal coping best predicted perceived stress in a linear regression model. Overall, passive avoidant coping styles (i.e., resignation and withdrawal), but not active avoidant coping style (i.e., diversion), were related to perceived stress. These results support the continued study of multiple aspects of avoidant coping styles as well as the avatar task to increase our understanding of the maladaptive effects of excessive avoidance in the face of stress.

## Introduction

When faced with an aversive situation, individuals differ in how they seek to reduce feelings of stress through coping. Some forms of coping are adaptive and lead to resilience in the face of stress while other forms of coping are maladaptive and may result in post-traumatic stress disorder (PTSD) or anxiety disorders (Dymond, 2019; Zoellner et al., 2020). Early work defined coping as involving approach or avoidance coping strategies (Folkman & Lazarus, 1980; Moos & Schaefer, 1984). Approach coping is defined as actively moving towards a stressor in order to seek information, social support, plan ahead, and attempt to solve the problems (Finset et al., 2002). Approach coping can also involve vigilance (Krohne, 1993) in that person deals with stress by increased attention and processing of aversive information. Unlike approach coping, avoidance coping is multidimensional. Avoidance coping has been defined as a passive coping strategy in which an individual disengages from a stressor or as an active coping strategy in which an individual turns away from or seeks to escape from a stressor (Folkman & Lazarus, 1988). Feifel & Strack (1989) also differentiated two similar two aspects of avoidance, avoidance and resignation. In addition, avoidance coping involves cognitive/emotional strategies to reduce thoughts or feelings such as mental disengagement or denial, or behavioral attempts to physically remove one’s self from an aversive situation.

Many coping styles fit into these categories of being adaptive or maladaptive and involving approach or avoidance coping. For example, a widely used inventory for the assessment of coping styles is the Brief Coping Orientation to Problems Experienced (Brief COPE; Carver, 1997) which measures fourteen coping styles that have more recently been categorized as adaptive or maladaptive as well as avoidant and non-avoidant coping styles. Kasi and colleagues (2012) grouped coping styles from the Brief COPE as adaptive (i.e., active coping, acceptance, emotional support, humor, instrumental support, positive reframing, religion) or maladaptive (i.e., behavioral disengagement, denial, self-blame, self-distraction, substance use, and venting). Subsequent analyses of the Brief Cope by Baumstarck and colleagues (2017) revealed an avoidance factor which consisted of behavioral disengagement, self-distraction, substance use, denial, and self-blame and three other non-avoidant factors involving social support, problem solving, and positive thinking. Based on this avoidant factor, a computer-based avatar task designed to assess avoidant personality temperaments was used to examine avoidant and non-avoidant coping styles as measured by the Brief COPE. Allen & Myers (2019) reported that performance on this task had a significant positive relationship with an aggregate score for the avoidant coping styles and a significant negative relationship with an aggregate score for the non-avoidant coping styles.

This avatar task was designed by Myers et al. (2016a) to assess behavioral inhibition or BI which is defined as a tendency to withdraw from unfamiliar individuals or situations (Kagan, Reznick & Snidman, 1987; Morgan, 2006). The avatar task was put forth as an alternative to paper and pencil self-report measures of BI such as the Adult Measure of Behavioural Inhibition (AMBI; Gladstone & Parker, 2005). Self-report inventories have several limitations. One obvious limitation is the potential for response bias and demand characteristics (McCambridge, deBruin & Witton, 2012). Myers and colleagues (2016a) suggested that some participants may (consciously or unconsciously) understate inhibited behavior in order to appear well-adjusted or conform to a positive view of self. For example, professionals, such as emergency workers, who develop PTSD while on the job may avoid participation in research with PTSD questionnaires due to fear of losing their jobs (Clohessy & Ehlers, 1999). This tendency in some individuals to bias their responses based on their avoidant tendencies and anxiety may be an attempt to avoid negative evaluation. Fear of negative evaluation has been linked to several forms of avoidance including social avoidance (Collins et al., 2005) and trait anxiety (Turner, McCanna & Beidel, 1987). Trait anxiety has been linked to behavioral inhibition (Caulfield, McAuley & Servatius, 2013) which is the personality temperament the avatar task was originally designed to assess. Rather than asking for participants to say what they would do, Myers et al. (2016a) developed the avatar task in which participants indicated how they would act when interacting with strangers in novel situations. In this task, the participant selected an onscreen character (“avatar”) which he/she guided through several scripted scenarios involving social interactions with strangers.

Performance on the avatar task had a strong positive relationship to participants’ scores on the AMBI (Myers et al., 2016a). In subsequent work, harm avoidance (HA), a tendency to respond strongly to aversive stimuli and learn to avoid punishment, novelty, and non-reward (Nixon & Parsons, 1989) was examined. HA as measured by the Tridimensional Personality Questionnaire (TPQ; Cloninger, Przybeck & Svrakic, 1991) was found to have a moderate positive relationship to performance on the avatar task (Allen, Jameson & Myers, 2017). Allen (2018) also reported that performance on the avatar task was related to behavioral, but not cognitive, avoidance as measured by the Cognitive Behavioral Avoidance Scale (CBAS, Ottenbreit & Dobson, 2004). Most recently, Allen & Myers (2019) reported that performance on avatar task had a significant positive relationship with avoidant coping styles and a significant negative relationship with non-avoidant coping styles as measured by the Brief COPE. Taken together, these findings support the idea that performance on the avatar task is related to several avoidant personality temperaments as well as provide some preliminary evidence that the avatar task can differentiate avoidant and non-avoidant coping styles.

Given that the avatar task has been found to differentiate avoidance and non-avoidant coping styles as measured by the Brief COPE, the current study sought to investigate avoidant coping as measured with another measure, the Brief Approach/Avoidance Coping Questionnaire (BACQ; Finset et al., 2002). The BACQ measures cognitive, socio-emotional, and behavioral coping styles which are divided into an avoidant and approach coping subscale. In addition, avoidant coping is further divided into active avoidant coping (i.e., diversion) and passive avoidant coping (i.e., resignation and withdrawal).

One unresolved issue with coping styles is how approach and avoidance coping are related to each other. There are mixed findings which indicate that approach and avoidance subscales may be negatively related (Miller, 1987), unrelated (Feifel & Strack, 1989), or positively related (Finset & Andersson, 2000). In addition, Carver and colleagues (1989) reported that the coping constructs connected to problem solving (i.e., active coping and planning) are negatively correlated with problem avoidance strategies (i.e., behavioral disengagement and denial). In developing the BACQ, Finset and colleagues (2002) put forth that approach and avoidance are not mirror images of each other. This conclusion was supported by Polman and colleagues (2010) who found that the relationships between approach coping and the avoidant subdimensions of diversion coping and resignation and withdrawal coping differ. Specifically, approach coping had a moderate negative relationship with resignation and withdrawal coping but a weak positive relationship with diversion coping. Diversion coping and resignation and withdrawal coping also had only a weak positive relationship with each other. The current study sought to continue this line of research to explore how approach and avoidance coping as measured by the BACQ relate to each other, to avoidant and non-avoidance coping styles as measured by the Brief COPE, and to performance on the avatar task.

Another issue explored in the current study is how avoidance and approach coping are related to undesirable outcomes such as stress or psychopathology. Overall, approach coping is viewed as being more adaptive than avoidance coping (Snyder & Pulvers, 2001). For example, Tiet et al. (2006) reported found that approach coping predicted improved social functioning of patients with chronic PTSD while passive coping strategies such as denial and disengagement were related to distress and negative affectivity (Rohde et al., 1990; Carver et al., 1993; Smari et al., 1997). The maladaptive effects of avoidant coping include clinical psychopathologies such as major depression, panic attacks, (Haaga et al., 2004; Ottenbreit & Dobson, 2004), and PTSD (Haaga et al., 2004; Ottenbreit & Dobson, 2004; Shipherd & Beck, 2005; Ullman et al., 2007; Pineles et al., 2011). PTSD has been viewed as an imbalance of approach-avoidance systems (Stein & Paulus, 2009) and avoidance is a symptom criterion for PTSD in the DSM-5 (American Psychiatric Association, 2013). There is some limited evidence that performance on the avatar task is positively correlated with PTSD avoidant symptom severity in a sample of Veterans (Myers et al., 2016b). However, the relationship of performance on the avatar task to stress in non-clinical populations has not been investigated. Therefore, the current study included the Perceived Stress Scale (PSS; Cohen, Kamarck & Mermelstein, 1994) which is the most widely used measure of the perception of stress and has been utilized with both clinical (Stanley et al., 2011; Wahbeh & Oken, 2013) and non-clinical samples (Deckro et al., 2002; Polman, Borkoles & Nicholls, 2010). In addition, previous work has examined the relationships of perceived stress with coping styles measured with both the Brief COPE and the BACQ. Karekla & Panayiotou (2011) reported that the Brief COPE subscales of active coping, behavioral disengagement, denial, emotional support, instrumental support, self-blame self-distraction, and venting were positively correlated with perceived stress while positive reframing was negatively correlated with PSS scores. The subscales that were positively correlated to perceived stress included four out of the five avoidance coping styles (with the exception of substance use) identified by Baumstarck and colleagues (2017) which were subsequently found to be positively related to performance on the avatar task by Allen & Myers (2019). In addition, Polman and colleagues (2010) found that perceived stress had a weak negative relationship with approach coping and a moderate positive relationship with avoidance coping (both diversion coping and resignation and withdrawal coping) as measured by the BACQ. Avoidance has also been found to moderate the relationship between anxiety sensitivity and perceived stress (Bardeen, Fergus & Orcutt, 2013) as well as to mediate the relationship between traumatic events and psychological distress (Batten, Follette & Aban, 2002; Marx & Sloan, 2002). Overall, there is increasing evidence that the PSS is a valid measure of stress in the context of avoidant coping which supports the continued investigation of the relationship between perceived stress, the Brief COPE, the BACQ, and performance on the avatar task in the current study.

### Hypotheses

The first aim of the current study was to compare the relationships of approach and avoidance coping as measured by the BACQ to avoidant and non-avoidant coping styles as measured by the Brief COPE as well as performance on the avatar task. Based on the findings of Polman and colleagues (2010), it was hypothesized that approach coping would have a negative relationship to avoidance as measured by the Brief COPE and the avatar task and have a positive relationship to non-avoidant coping as measured by the Brief COPE. Also based on the findings of Polman and colleagues (2010) as well as the findings of Allen & Myers (2019), it was hypothesized that scores on the avatar task would be positively correlated to avoidance scores and negatively correlated to approach scores on the BACQ. More specifically, performance on the avatar task was hypothesized to be positively related to both diversion coping and resignation and withdrawal coping.

The second aim of the current study was to examine the relationships between approach and avoidance coping as measured by the BACQ, avoidance and non-avoidance coping as measured by the Brief COPE, and performance on the avatar task with the outcome of perceived stress as measured by the PSS. Based on the relationship of perceived stress and avoidance coping as measured by the BACQ (Polman, Borkoles & Nicholls, 2010), it was hypothesized that avoidance coping as well as scores on the avatar task would be positively correlated to scores on the perceived stress while approach and non-avoidance coping would be negatively associated with perceived stress.

## Material and Methods

### Participants

The current sample was comprised of one hundred undergraduates who voluntarily completed the study for partial research credit for an introductory psychology course. All procedures were approved by the Institutional Review Board at University of Northern Colorado (approval # 799609-4). Following written informed consent, participants completed a short demographic questionnaire in which they indicated their gender, age, years of education, and race/ethnicity. Participants included 70 females and 30 males with a mean age of 18.8 years (SD = 1.3, range 18–25) and a mean education level of 12.8 years of schooling (SD = 1.1, range 12–17). The sample was mainly Caucasian (*n* = 49), followed by Hispanic (*n* = 25), multi-racial (*n* = 18), African-American (*n* = 6), and East Asian (*n* = 1).

### Instruments

The Brief Coping Orientation to Problems Experienced (Brief COPE; Carver, 1997) contains twenty-eight items with two items for each of the following coping styles: acceptance, active coping, behavioral disengagement, denial, emotional support, humor, instrumental support, planning, positive reframing, religion, self-blame, self-distraction, substance use, and venting. Each item asked participants to rate how much they coped with a hardship in life by using a specific coping style. An example item is “I have been turning to work or other activities to keep my mind off things”. Participants indicated their responses on a 4-point Likert-type scale with choices ranging from don’t do this at all (1 point), do this a little bit (2 points), do this a medium bit (3 points), and do this a lot (4 points). The Brief COPE has satisfactory test-retest reliability and convergent and divergent validity (Muller & Spitz, 2003; Cooper, Katona & Livingston, 2008). In addition to the overall inventory, the two items for each subscale (Carver, 1997; Muller & Spitz, 2003; Cooper, Katona & Livingston, 2008; Yusoff, Low & Yip, 2010) as well as the five subscales forming the aggregate avoidance score (Baumstarck et al., 2017) have acceptable internal reliability. The Brief COPE has satisfactory psychometric properties (Muller & Spitz, 2003) including test-retest reliability and convergent and divergent validity in a sample of undergraduates (Cooper, Katona & Livingston, 2008). Studies have indicated that the two items for each subscale have acceptable internal validity as measured by Cronbach’s alphas ranging from .57 to .89 (Carver, 1997; Cooper, Katona & Livingston, 2008; Muller & Spitz, 2003; Yusoff, Low & Yip, 2010). Baumstarck et al. (2017) reported acceptable internal reliability between the five subscales forming the aggregate avoidance score (Cronbach’s alpha = .64). The current study had acceptable internal consistency as indicated by a Cronbach’s alpha of .89 for the avoidant aggregate score and .70 for the non-avoidant aggregate score.

The Brief Approach/Avoidance Coping Questionnaire (BACQ; Finset et al., 2002) includes two subscales, approach and avoidance, each of which consists of six items. Participants are asked to indicate how they usually cope with problems and illness using a five-point Likert-type scale ranging from “agree completely” to “disagree completely”. An example of an approach item is “I make an active effort to find a solution to my problems”. An example of an avoidance item is “I put my problems behind me by concentrating on something else”. The avoidance factor was further delineated into a diversion factor (i.e., active coping) and resignation and withdrawal factor (i.e., passive coping). The BACQ has adequate psychometric properties as indicated by a Cronbach’s alpha of .68 for the overall scale .59 for the approach subscale, .55 for the avoidance subscale as well as .53 for the resignation and withdrawal factor and .66 for the diversion factor as first reported by Finset et al. (2002) and subsequently supported by Polman and colleagues (2010). The current study had a Cronbach’s alpha of .66 for the approach scale and .71 for the avoidant scale (diversion = .72; resignation and withdrawal =.69).

The Perceived Stress Scale (PSS; Cohen, Kamarck & Mermelstein, 1994) consists of ten items which ask participants to indicate using a five-point Likert-type scales how often they felt and thought a certain way during the last month (never, almost never, sometimes, fairly often, or very often). An example item is “In the past month, how often have you felt unable to control the important things in your life?”. Cronbach’s alpha for the 10-item version of the PSS ranged from .78 –.92 (Cole, 1999; Karekla & Panayiotou, 2011; Lee, 2012). The current study had a comparable Cronbach’s alpha of .63 for the PSS.

### Computer-based task

**Figure 1:**
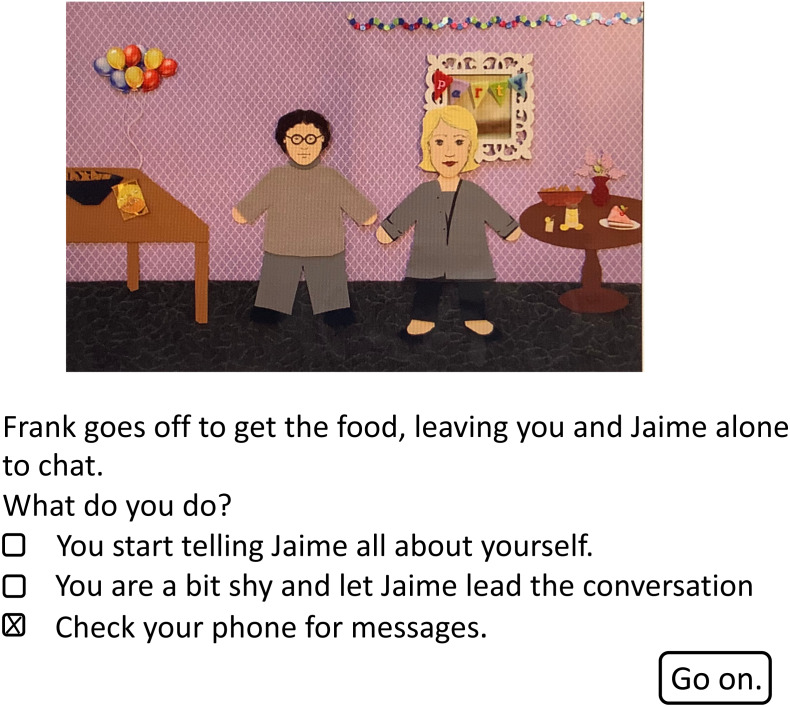
A sample screen capture from the avatar task. In the first scenario, the avatar was invited to a party by her cousin and left alone to talk to someone that she did not know. At this point, the avatar has a choice between a non-avoidant response, an intermediate response, and an avoidant response. Here the participant has chosen the avoidant response.

The computer-based task has been previously described (Myers et al., 2016a) and is available for download from the Open Science Framework (http://www.osf.io/zf3jv). At the start of the task, the researcher read instructions to the participant in which he/she was asked to select an avatar to represent him/her in the task and to think of the avatar as himself/herself and to make decisions like he/she would in real life. Participants then selected from a set of eight possible avatars with varying skin color, hair color, and hair length. The participant then guided his/her avatar through a scenario in which the avatar attended a party where he/she didn’t know anyone followed by a second scenario in which the avatar volunteered to work on a charity building project with a group of strangers. The two scenarios included twenty decision points with a short text description and a still image showing the avatar experiencing this event, as shown in Fig. 1. At each decision point, the participant chose from three response options consisting of a relatively avoidant, a relatively non-avoidant, and an intermediate response. For example, in the first scenario, the avatar is invited to a party by his/her cousin but the cousin is not there when he/she arrives at the house. The choices are to enter the party (relatively non-avoidant), wait outside for the cousin to arrive (relatively avoidant), or call the cousin to see when he will get there (intermediate). The participant received two points for choosing the avoidant option, one point for the intermediate option, and zero points for the non-avoidant option. Total scores could range from 0 (least avoidant) to 40 (most avoidant). Scores and points were not visible to the participants. All participants experienced the same scripted sequence of events and response options regardless of their individual responses. The current sample produced excellent internal reliability for the avatar task as indicated by a Cronbach’s alpha of .89 which is consistent with prior reports (Myers et al., 2016a; Myers et al., 2016b; Allen & Myers, 2019).

### Procedures

The study was completed by individual participants in a small room in the presence of an individual researcher. The order of the avatar task and paper and pencil inventories were counterbalanced across participants. The avatar task took about 10 min to complete. The entire experiment including the paper and pencil inventories and the avatar task took less than 30 min.

### Data analysis

Total scores for the Brief COPE were summed across all 28 items. Individual coping style scores were also calculated. An aggregate avoidance and non-avoidance score were calculated for each participant in which the five coping styles of behavioral disengagement, denial, self-blame, self-distraction, and substance use were summed together as an aggregate avoidance factor while the remaining nine coping styles were summed together as an aggregate score for the non-avoidance coping styles.

Inventory scores and scores on the avatar task were analyzed for normality with the Kolmogorov Smirnov Test of Normality. Based on all inventory results being normally distributed, Pearson’s product moment correlations were calculated between the BACQ approach and avoidance scores (including diversion coping and resignation and withdrawal coping subscales), the aggregate avoidance and non-avoidance scores from the Brief COPE, scores on the avatar task, and the scores on the PSS. Group differences including gender effects were analyzed with an independent measures *t*-test. Stepwise linear regressions were calculated with predictors of the scores on the BACQ approach and avoidance scales including the diversion and resignation subscales and Brief COPE avoidant and non-avoidant aggregate scores. Dependent variables were scores on the avatar task and scores on the PSS.

## Results

### Mean scores and gender comparisons

The mean scores for inventories and the avatar task along with female and male means are shown in Table 1. Overall, there were no significant gender differences for any of the inventory scales or subscales. There was also no gender effect for scores on the avatar task. Thus, gender was not included in subsequent analyses.

**Table 1:** Inventory scores gender comparison.

	Overall mean (sd)	Female mean (sd)	Male mean (sd)	Gender significance level
BACQ Approach	22.0 (4.1)	21.7 (4.5)	22.6 (4.5)	ns
BACQ Avoidance	16.9 (4.3)	17.1 (4.2)	15.8 (4.3)	ns
Diversion	9.3 (2.7)	9.5 (2.6)	8.6 (3.0)	ns
Resignation & Withdrawal	7.4 (2.6)	7.6 (2.5)	7.0 (2.7)	ns
Brief COPE Avoidance Aggregate	20.4 (3.7)	20.4 (3.7)	20.6 (3.2)	ns
Brief COPE Non-Avoidance Aggregate	52.1 (7.7)	51.5 (7.8)	53.5 (7.7)	ns
Avatar Task Score	19.0 (6.1)	19.4 (5.6)	18.0 (6.5)	ns

### Relationships between the BACQ and the brief COPE

The relationships between the scores on the BACQ, and the Brief COPE are summarized in Table 2. Overall, avoidance coping as measured by the BACQ, had a weak negative relationship to approach coping. More specifically, approach coping had a moderate negative relationship to resignation and withdrawal coping, but no significant relationship to diversion coping. BACQ avoidance coping also had a strong positive relationship to both diversion coping and resignation and withdrawal coping.

**Table 2:** Relationships of avoidance measures.

	Avoidant coping (BACQ)	Diversion coping (BACQ)	Resignation & withdrawal coping (BACQ)	Approach coping (BACQ)	Avoidant coping (Brief COPE)	Non-Avoidant coping (Brief COPE)	Performance on the Avatar Task	Perceived stress scale (PSS)
Avoidant Coping (BACQ)	–							
Diversion Coping (BACQ)	.82^**^	–						
Resignation & Withdrawal Coping (BACQ)	.79^**^	.31^*^	–					
Approach Coping (BACQ)	-.20^*^	-.02	-.31^*^	–				
Avoidant Coping (Brief COPE)	.58^**^	.45^**^	.49^**^	-.23	–			
Non-Avoidant Coping (Brief COPE)	-.03	.09	-.20	.39^**^	.02	–		
Performance on the Avatar Task	.47^**^	.36^**^	.37^**^	-.31^*^	.35^**^	-.16	–	
Perceived Stress Scale (PSS)	.48^**^	.15	.67^**^	-.38^**^	.53^**^	-.25^*^	.38^**^	–

**Notes.**

* *p* < .05.

** *p* < .001.

BACQ avoidance coping had a moderate positive relationship with the avoidant aggregate score from the Brief COPE and a non-significant relationship with the non-avoidant aggregate score from the Brief COPE. The same relationships were observed between diversion coping and resignation and withdrawal coping and the avoidant and non-avoidant aggregate scores from the Brief COPE. BACQ approach coping had a moderate positive relationship with the avoidant aggregate score from the Brief COPE and a weak negative relationship with the non-avoidant aggregate score from the Brief COPE.

### Relationships to performance on the avatar task

The relationships between scores on the avatar task and coping styles from the BACQ and Brief COPE are also summarized in Table 2. Overall, avoidant coping from the BACQ and the avoidant aggregate scores from the Brief COPE had a moderate positive relationship to performance on the avatar task. In addition, both diversion coping and resignation and withdrawal coping had moderate positive relationships with scores on the avatar task. Approach coping as measured by the BACQ had a moderate negative relationship to scores on the avatar task while the non-avoidant aggregate score from the Brief COPE had a non-significant negative relationship to scores on the avatar task.

As shown in Table 3, backward stepwise linear regression with the BACQ coping styles (approach, avoidance, diversion, and resignation and withdrawal coping) and avoidant and non-avoidant aggregate scores from the Brief COPE as predictors revealed that avatar scores could be best predicted by a model that included approach, diversion, and resignation and withdrawal coping from the BACQ (R = .51, R^2^ = .232, F (3, 96) = 10.97, *p* <.001).

**Table 3:** Details on the regression analyses predicting performance on the avatar task and perceived stress.

Variables	Standardized *β*	b	Standard Error	*p* Value	R^2^
**Best predictor of performance on the avatar task**					
Approach Coping	-0.243	-0.347	0.132	0.01	0.232
Diversion	0.278	0.601	0.200	0.003	
Resignation and Withdrawal	0.214	0.489	.222	0.03	
Constant		17.307	3.900	<0.001	
**Best predictor of perceived stress**					
Resignation and Withdrawal	0.345	0.440	0.121	<0.001	0.130
Constant		19.966	0.949	<0.001	

### Relationships to perceived stress

The relationships between perceived stress and the coping styles from the BACQ, and Brief COPE, as well as scores on the avatar task are also summarized in Table 2. Avoidance coping as measured by the BACQ and the avoidant aggregate score from the Brief COPE had a moderate positive relationship with perceived stress. While resignation and withdrawal coping had a strong positive relationship to perceived stress, diversion coping had a non-significant relationship. Approach coping as measured by the BACQ and non-avoidant aggregate score from the Brief COPE had a moderate negative relationship to perceived stress. Performance on the avatar task also had a moderate positive relationship to perceived stress.

As shown in Table 3, backward stepwise linear regression with the BACQ coping styles (approach, diversion, and resignation and withdrawal), avoidant and non-avoidant aggregate scores from the Brief COPE, and scores from the avatar task as predictors revealed that a model which only included resignation and withdrawal coping was the single best predictor of perceived stress (R = .36, R^2^ = .130, F (1, 98) = 13.23, *p* <.001).

## Discussion

The current study had two aims. The first aim was to investigate the relationships of avoidance and approach coping styles as measured by the BACQ with avoidant and non-avoidant styles as measured by the Brief COPE and performance on the avatar task. The second aim was to explore the relationships of perceived stress as measured by the PSS with avoidance and approach coping styles as measured by the BACQ, avoidant and non-avoidant styles as measured by the Brief COPE, and performance on the avatar task.

First, avoidant coping measured both by the BACQ and by the avoidant aggregate score from the Brief COPE had a weak negative relationship with approach coping on the BACQ. More specifically, the avoidance subdimension of resignation and withdrawal had a moderate negative relationship to approach coping which fits with the findings of Polman and colleagues (2010) that indicated a weak negative relationship between resignation and withdrawal coping and approach coping. The current study also found no significant relationship between diversion coping and approach coping which differed from the report of a weak positive relationship (*r* = .12) between diversion and approach coping (Polman, Borkoles & Nicholls, 2010). However, in either case, diversion avoidant coping did not have a significant negative relationship with approach coping. The finding of only a weak negative relationship between approach and avoidance confirms the conclusion of Finset and colleagues (2002) that approach and avoidance coping are not simply opposing coping styles. The finding that withdrawal and resignation has a much stronger negative relationship to approach than diversion also supports the idea that avoidance in not a unidimensional measure (Folkman & Lazarus, 1988) and should be examined with multi-dimensional approaches.

Avoidant coping as measured by the BACQ had a moderate positive relationship to the avoidant aggregate score of the Brief COPE and a non-significant relationship to the non-avoidant aggregate score of the Brief COPE. More specifically, both resignation and withdrawal coping and diversion coping had a moderate positive relationship to the avoidant aggregate score. However, resignation and withdrawal coping had a significant negative relationship with the non-avoidant aggregate score of the Brief COPE while diversion had a non-significant relationship. These findings fit with the results of Finset and colleagues (2002) which compared approach and avoidance as measured by the BACQ with a subset of scales from the COPE (Carver, Scheier & Weintraub, 1989) which was an earlier version of the Brief COPE used in the current study. Specifically, the COPE scales of active coping, seeking emotional social support, and positive reinterpretation were characterized as approach coping while behavioral and mental disengagement were characterized as avoidance coping. The COPE approach scales had moderate positive relationships with BACQ approach coping, moderate negative relationships with resignation and withdrawal coping, and weak negative relationships with diversion coping. The COPE avoidance scales had moderate positive relationships with resignation and withdrawal coping. Mental disengagement, but not behavioral disengagement, had a moderate positive relationship with diversion coping. Finset and colleagues (2002) concluded that while approach and passive avoidance (i.e., resignation and withdrawal) are opposing poles of the same factor, diversion (i.e., active avoidance) is a separate factor in the BACQ. This conclusion matches the results of the current study in that resignation and withdrawal coping and diversion coping differ in their relationships to approach coping.

The current study also included a computer-based avatar task that has been found to have a moderate positive relationship with avoidant coping as measured by an aggregate score on the Brief COPE (Allen & Myers, 2019). The current work replicated this finding and also revealed a moderate positive relationship between scores on the avatar task and the avoidance scale of the BACQ. In addition, the BACQ also allowed for the comparison of performance on the avatar task to more specific forms of avoidance coping, mainly active (diversion) and passive (resignation and withdrawal) avoidant coping. Diversion coping and resignation and withdrawal coping had similar moderate positive relationships to performance on the avatar task. The finding of similar relationships for active and passive avoidance was somewhat surprising given that avatar task was designed based on behavioral inhibition. However, several inhibited choices in the avatar task involved active avoidance such as engaging in other activities like checking for cell phone messages or making phone calls as alternatives to interacting with strangers. There are also a few inhibited choices in the avatar task such as staying home and relaxing or waiting for someone you know to show up that could be interpreted as passive avoidance. In addition, scores on the avatar task had a moderate negative relationship with the approach scale of the BACQ. While performance on the avatar task was found to have a negative relationship with non-avoidant coping styles on the Brief COPE (Allen & Myers, 2019), the negative relationship with approach coping is a novel finding that provides further evidence for the selectivity of the avatar task to measure avoidance. This current finding of a negative relationship between avatar scores and approach coping is consistent with the prior finding of Allen, Jameson & Myers (2017) in which scores on the avatar task were negatively correlated to novelty seeking (*r* =  − 0.39) as measured by the Tridimensional Personality Questionnaire (TPQ; (Cloninger, Przybeck & Svrakic, 1991)). A tendency against approaching and dealing directly with a stressor fits with the overall tendency of harm avoidant and behavioral inhibited individuals to avoid or withdraw from novelty or uncertainty. (Kagan, Reznick & Snidman, 1987; Morgan, 2006). The finding that scores on the avatar task had no significant relationship to the non-avoidance aggregate score on the Brief COPE differed from the previous finding that non-avoidant aggregate score of the Brief COPE had a negative relationship with performance on the avatar task (Allen & Myers, 2019). This non-avoidant aggregate consisted of scores for active coping, acceptance, emotional support, humor, instrumental support, planning, positive reframing, religion, and venting. Since some of these coping styles (e.g., humor, religion, and venting) do not involve approach coping in that they do not involve facing the stress/trauma directly, the non-avoidant coping styles from the Brief COPE may not be directly comparable to approach coping as measured by the BACQ. Overall, performance on the avatar task may have either no relationship or at best a weak negative relationship to non-avoidant coping styles, but does have a moderate negative relationship to approach coping as measured by the BACQ. These findings were further confirmed by a regression analysis of the BACQ and Brief COPE measures which indicated that the combination of approach, diversion, and resignation and withdrawal coping as measured by the BACQ was the best predictor of performance on the avatar task.

The second aim of the current study was to explore the relationships of perceived stress as measured by the PSS to avoidance and approach coping as measured by the BACQ and avoidance coping as measured by the Brief COPE. Avoidance coping as measured by the BACQ and the Brief COPE had similar moderate positive relationships to perceived stress. This finding agrees with prior work (Polman, Borkoles & Nicholls, 2010; Karekla & Panayiotou, 2011) in which positive relationships were found between avoidant coping styles and scores on the PSS. The current study also indicated that approach coping had a moderate negative relationship with perceived stress which fits with the findings of Polman and colleagues (2010). These findings are also supported by studies comparing the COPE/Brief COPE avoidant scales to the PSS. Litman (2006) found that avoidant coping styles (behavioral disengagement, denial, substance use, and mental disengagement) from the COPE (Carver, Scheier & Weintraub, 1989) were positively correlated with avoidance, anxiety, and depression and were negatively correlated or unrelated to approach, extraversion, and curiosity. More specifically, resignation and withdrawal avoidance coping, but not diversion avoidance coping, had a strong positive relationship to perceived stress. These findings were further confirmed by a regression analysis of the possible predictors of perceived stress which indicated that a model including only resignation and withdrawal was the single best predictor of this outcome. The current findings are consistent with other previous studies which have suggested that passive coping is associated with anxiety (Roy-Byrne et al., 1992; Rodrigue et al., 1993), and distress and negative affectivity (Carver et al., 1993; Smari et al., 1997). More specifically, these current results also fit with those of Polman and colleagues (2010) in which resignation and withdrawal had a moderate positive relationship to perceived stress and other undesirable outcomes such as exhaustion and disengagement while diversion only had a weak positive relationship to perceived stress. Taken together, the current and prior findings contribute to a growing literature which identifies passive avoidance coping, rather than active avoidance coping, as a maladaptive response to stressful or traumatic situations which ultimately may lead to poor outcomes.

Performance on the avatar task also had a moderate positive relationship with perceived stress which indicates that the avatar task can identify stress in non-clinical populations. The finding is consistent with the work of Myers and colleagues (2016b) which reported that scores on the avatar task have a moderate positive relationship to PTSD symptoms as measured by the PTSD checklist (Blanchard et al., 1996). However, this study with Veterans self-reporting PTSD symptoms did not examine coping styles. Future work with the avatar task in clinical populations should include measures of coping style to further investigate how various forms of coping may positively or negatively influence avoidant symptoms of PTSD.

The current findings with avoidance coping, the avatar task, and perceived stress may also be applicable to the study of therapies that target avoidance as a treatment for psychopathologies such as anxiety disorders, PTSD, and depression. Several different types of psychotherapy and cognitive–behavioral interventions are designed to reduce avoidant behaviors, reactions, and thoughts (Abramowitz, Deacon & Whiteside, 2013; Dobson & Dobson, 2017) and reduce symptoms of anxiety (Borkovec et al., 2002) and depression (Lejuez, Hopko & Hopko, 2001; Martell, Addis & Jacobson, 2001; Hopko et al., 2003). Specifically, psychotherapies including acceptance and commitment therapy (ACT) target experiential avoidance or EA (Tyndall et al., 2019). EA is defined as attempts to avoid aversive thoughts, feelings, memories, physical sensations as well as avoiding aversive events or stimuli that produce these internal experiences (Hayes et al., 1996) and has been to found to be related to avoidance coping styles. For example, Karekla & Panayiotou (2011) tested the relationships between EA and avoidance coping styles as measured by the Brief COPE and found that that higher levels of EA were related to emotion-focused and avoidant coping styles such as behavioral disengagement, denial, emotional support, self-blame, self-distraction, and venting Conversely, lower levels of EA were associated with more adaptive coping styles such as positive framing and acceptance. In addition, EA is related to perceived stress (Karekla & Panayiotou, 2011), moderates the relationship between anxiety sensitivity and perceived stress (Bardeen, Fergus & Orcutt, 2013), and mediates the relationship between traumatic events and psychological distress (Batten, Follette & Aban, 2002; Marx & Sloan, 2002). Experiential avoidance also moderates post-traumatic stress in that it tends to produce stress rather than growth (Kashdan & Kane, 2011).

The current study also sought to extend a series of studies with the avatar task that examined its ability to assess avoidant personality temperaments such as behavioral inhibition (Myers et al., 2016a), harm avoidance (Allen, Jameson & Myers, 2017), cognitive and behavioral avoidance (Allen, 2018) as well as avoidant coping styles (Allen & Myers, 2019) and PTSD symptoms (Myers et al., 2016b). The current work sought to address several limitations of recent work. For example, Allen & Myers (2019) examined avoidant coping styles but this study only utilized one measure of coping styles, the Brief COPE. The current study addressed this limitation by the inclusion of the BACQ which revealed the positive relationship between avoidance coping including both active (i.e., diversion) and passive (i.e., resignation and withdrawal) avoidant coping and performance on the avatar task. Future work could extend this work to include other measures of avoidant coping styles such as the Coping Inventory for Stressful Situations (CISS; Endler & Parker, 1999) as well as measures of experiential avoidance such as the Acceptance and Action Questionnaire II (AAQ II; Bond et al., 2011) or the Multidimensional Experiential Avoidance Questionnaire (MEAQ; Gamez et al., 2011). To the author’s knowledge, the relationship of EA has not been tested with approach and avoidance coping as measured by the BACQ or with the avatar task. Future work could examine the effectiveness of therapies designed to reduce EA to not only reduce avoidance coping but increase approach coping. These future studies could address a limitation of the current study in that it only involved a non-clinical sample of undergraduates. Future work can utilize clinical populations, especially those individuals receiving therapies designed to reduce EA as well as individuals who exhibit PTSD symptoms.

## Conclusions

The current study explored the relationships between avoidance and approach coping as measured with the BACQ, avoidance coping as measured by the Brief COPE, and a computer-based avatar task as well as their relationships with perceived stress. The current study supports the concept that avoidance and approach coping have only a weak negative relationship and are not assessing opposing coping styles. This weak negative relationship comes about, in part, due to the multiple dimensions of avoidance coping in which passive avoidance coping (resignation and withdrawal) has a moderate negative relationship with approach coping while active avoidance coping (diversion) has no significant relationship. In addition, while avoidance coping is positively correlated to perceived stress, this relationship is mainly due to the strength of the relationship with resignation and withdrawal coping rather than diversion coping. The current results also support the avatar task as an assessment applicable for assessing to avoidance coping, including both passive and active avoidance coping. The current study also found novel evidence that performance on the avatar task is positively related to perceived stress which supports the further application of this task to the study of PTSD and anxiety disorders. Overall, the current findings indicate the importance of continued focus on the role of passive avoidance coping styles such as resignation and withdrawal in producing maladaptive outcomes including stress, anxiety disorders, and PTSD.

##  Supplemental Information

10.7717/peerj.11265/supp-1 Supplemental Information 1 Demographic information and raw scores for the avatar task, Brief COPE, BACQ, and PSS

## Additional Information and Declarations

### Data Availability

The following information was supplied regarding data availability: The demographic data and inventory scores and scores on the avatar task, Brief COPE, BACQ, and PSS are available in the Supplemental File.
